# Monitoring Fatigue Damage of Orthotropic Steel Decks Using Nonlinear Ultrasonic Waves

**DOI:** 10.3390/ma17122792

**Published:** 2024-06-07

**Authors:** Jiahe Liu, Fangtong Zheng, Wei Shen, Dongsheng Li

**Affiliations:** 1 School of Civil Engineering, Dalian University of Technology, Dalian 116024, China; 1104060917@mail.dlut.edu.cn; 2 China Northeast Architectural Design & Research Institute Co., Ltd., Shenyang 110000, China; zhengfangtong@126.com; 3 State Key Laboratory of Featured Metal Materials and Life-Cycle Safety for Composite Structures, School of Civil Engineering and Architecture, Guangxi University, Nanning 530004, China; shenwei431@gxu.edu.cn; 4 State Key Laboratory of Coastal and Offshore Engineering, Dalian University of Technology, Dalian 116024, China

**Keywords:** orthotropic steel decks (OSDs), structural health monitoring, ultrasonic, fatigue, nonlinear parameters

## Abstract

Orthotropic steel decks (OSDs) are commonly used in the construction of bridges due to their load-bearing capabilities. However, they are prone to fatigue damage over time due to the cyclic loads from vehicles. Therefore, the early structural health monitoring of fatigue damage in OSDs is crucial for ensuring bridge safety. Moreover, Lamb waves, as elastic waves propagating in OSD plate-like structures, are characterized by their long propagation distances and minimal attenuation. This paper introduces a method of emitting high-energy ultrasonic waves onto the OSD surface to capture the nonlinear Lamb waves formed, thereby calculating the nonlinear parameters. These parameters are then correlated with the fatigue damage endured, forming a damage index (DI) for monitoring the fatigue life of OSDs. Experimental results indicate that as fatigue damage increases, the nonlinear parameters exhibit a significant initial increase followed by a decrease. The behavior is distinct from the characteristic parameters of linear ultrasound (velocity and energy), which also exhibit changes but to a relatively smaller extent. The proposed DI and fatigue life based on nonlinear parameters can be fitted with a Gaussian curve, with the R-squared value of the fitting curve being close to 1. Additionally, this paper discusses the influence of rib welds within the OSDs on the DI, whereby as fatigue damage increases, it enlarges the value of the nonlinear parameters without altering their trend. The proposed method provides a more effective approach for monitoring early fatigue damage in OSDs.

## 1. Introduction

Orthotropic steel decks (OSDs) form a composite structural system that is reinforced by welding top plates, longitudinal ribs, and transverse stiffeners. Originating in Germany during the 1930s [1], OSD technology has since been adopted worldwide, exemplified by its use in the Bronx–Whitestone Bridge in the United States, the Daishi Bridge in Japan, and the Humen Bridge in China [2]. The widespread popularity of OSDs can be attributed to several key advantages, including their lightweight nature, exceptional load-bearing capacity, minimal joint requirements, and ease of construction [3,4].

However, with the increasing service life and number of OSD bridges, it has become evident that OSDs are not without their issues [1,5]. For instance, they require complex welding details and are prone to intricate stress patterns during the initial design phase [6,7]. Over time, they are susceptible to the frequent passage of overweight vehicles, which significantly heightens the risk of fatigue damage, posing a direct threat to the overall safety of the bridge. The previously mentioned Bronx–Whitestone Bridge, Daishi Bridge, and Humen Bridge have all experienced fatigue-induced cracks [2], necessitating regular maintenance and inspections. Therefore, real-time and effective monitoring of the OSD’s fatigue life is crucial, especially for the early detection of fatigue damage.

Currently, various methods for identifying fatigue damage have been developed, including ultrasonic testing [2,8,9,10], strain measurement [11,12,13], acoustic emission [14], electro-mechanical impedance [6], infrared thermography [15], magnetic flux leakage [16], and prediction methods based on temperature and traffic data [17]. Strain measurement employs strain gauges to detect deformation at specific locations [13], while infrared thermography captures images based on temperature variations at crack sites [15]. Although infrared thermography and magnetic flux leakage methods provide a certain level of visual detection, their sensitivity in the early stages of crack and damage detection is insufficient, making it difficult to capture the appearance and development of small cracks in a timely manner. Prediction methods based on temperature and traffic data establish a nonlinear relationship between traffic information, temperature variations of the bridge deck, and the fatigue life of the OSD [17]; however, they rely on extensive historical data and complex algorithms. This makes them highly dependent on data completeness and model accuracy. In practical applications, the variability of traffic flow and environmental temperature often compromises the applicability and precision of these prediction models.

In contrast, ultrasonic testing requires fewer sensors and offers longer propagation distances [18], making it more promising for early fatigue prediction in OSDs [2,8,9,10]. Shi et al. [2,10] experimentally and numerically validated the fact that reflected Lamb waves can be used to monitor the geometric shape of cracks that are caused by fatigue in OSDs, highlighting the importance of sensor placement for effective monitoring. Gao et al. [8] utilized a pitch-catch sensor configuration to analyze the energy of ultrasonic backscattering, enabling the early warning of fatigue damage in welded steel plates. The aforementioned ultrasonic monitoring methods for fatigue damage rely on linear ultrasonic evaluation metrics, such as changes in the energy and velocity of scattered waves.

The early cracks in OSDs are better explained by contact acoustic nonlinearity theory [10], as linear velocity and energy tend to plateau during the initial stages of micro-damage [19], limiting their sensitivity to early fatigue damage. Nonlinear ultrasound, in contrast, is more sensitive in characterizing fatigue micro-damage. Within a certain amplitude range, nonlinear ultrasound generates higher harmonics through interaction with the material’s nonlinearity [20,21]. These harmonics arise from the coupling of the fundamental wave with microstructures, defects, and lattice dislocations within the material. Lee et al. [20] quantified the direction and length of cracks using nonlinear parameters through numerical simulation. These nonlinear parameters are not only applicable to steel plates but are also effective in assessing early micro-damage in aluminum plates [21] that has been caused by fatigue. Compared to ordinary steel or aluminum plates, OSDs feature U-ribs and weld seams. The presence of weld seams inevitably induces nonlinear effects [22], posing greater challenges for monitoring fatigue damage in OSDs. Therefore, investigating the applicability of nonlinear ultrasound technology for monitoring the fatigue life of OSDs is of significant interest.

This paper makes the following key contributions: it is the first to explore the interaction between nonlinear ultrasonics and fatigue cracks in OSDs, with particular attention to the impact of weld seams at the OSD ribs on nonlinear ultrasonic signals. A novel detection method is introduced, capable of quantitatively analyzing the remaining fatigue life of OSDs by fitting the DI based on nonlinear parameters to a Gaussian curve, which will be validated on seven OSD specimens. The method’s efficacy is thoroughly compared with traditional linear ultrasonic evaluation methods.

The structure of this paper is as follows: Section 2 introduces the evaluation methods and the preparation of the required materials, including the derivation of the DI based on nonlinear ultrasound, the fabrication of OSDs, fatigue testing, and ultrasound testing configurations; Section 3 presents the changes in linear and nonlinear ultrasound signals before and after OSD fatigue, along with the fitting analysis of DI and fatigue life; Section 4 compares the proposed method with linear ultrasound evaluation methods and examines the impact of weld seams on nonlinear parameters; and Section 5 concludes the paper.

## 2. Materials and Methods

### 2.1. Nonlinear Ultrasonics

**Figure 1:**
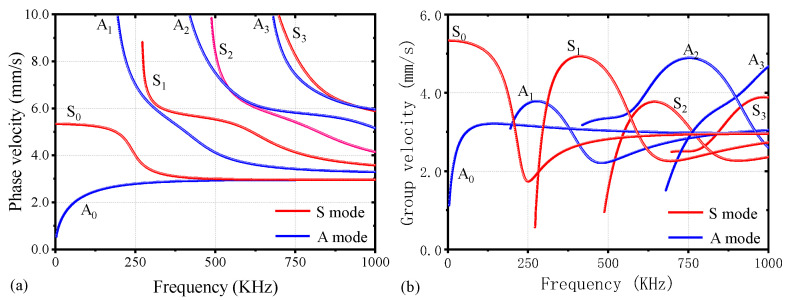
Lamb waves dispersion curves of OSDs: (**a**) phase velocity and (**b**) group velocity.

The British physicist Sir Horace Lamb first described Lamb waves in 1917 [23]. These waves can propagate in plate-like structures with parallel free boundaries, similar to steel plates. Lamb waves exhibit two fundamental modes—the symmetric mode (S-mode) and the antisymmetric mode (A-mode) [24]. In the S-mode, the plate vibrates simultaneously outward or inward, while in the A-mode, the vibrations on either side of the plate are opposite. Moreover, their properties vary with frequency. The relationship between the velocity and frequency of Lamb waves in OSDs can be determined through the Rayleigh–Lamb equation [25], as illustrated in Figure 1. It is evident that at any given frequency in OSD, both modes will be present. However, since the subsequent ultrasonic excitation is in an antisymmetric manner, the A-mode is expected to be the predominant mode of vibration [24].

Fatigue cracks within OSDs may exhibit an alternating open-and-close state under cyclic loads of a certain amplitude, a phenomenon vividly termed the “breathing effect” [20]. This effect results in local nonlinear interactions between the Lamb waves propagating in the OSD and the vicinity of the fatigue cracks, leading to waveform distortions and the emergence of second-order or higher-order harmonics. The internal stress endured by the contact surfaces of the breathing cracks can be represented using a simplified one-dimensional model, as follows:

(1) $$\sigma =E\varepsilon \left(1+\beta \varepsilon +\cdots \right)$$

where E(pa) is the modulus of elasticity; β represents the second-order elastic coefficient; and σ(pa) and ε denote stress and strain, respectively. By neglecting the higher-order terms beyond the second order in Equation (1) and utilizing the relationship between particle displacement and strain, substitution into the one-dimensional wave equation yields the following result [26]:

(2) $$\frac{{𝜕}^{2}u}{𝜕t^{2}}=c^{2}\frac{{𝜕}^{2}u}{𝜕x^{2}}+2c^{2}\rho \frac{𝜕u}{𝜕x}\frac{{𝜕}^{2}u}{𝜕x^{2}}$$

where u(m) represents displacement; $\rho (kg/m^{3})$ is the density of the medium; x(m) is the distance over which the wave propagates; t(s) stands for time; and c(m/s) is the wave speed. 

Based on perturbation theory [26], it is assumed that the displacement u is composed of a linear response, $u_{l}$, and a nonlinear response, $u_{nl}$. By synthesizing both the multiscale method and the trial solution method, as well as neglecting higher-order small quantities during the solution process, an approximate analytical solution to Equation (2) can be obtained, as follows:

(3) $$\;u=u_{l}+u_{nl}=U_{1}\cos\left(kx-\omega t\right)-U_{2}\sin\left[2\left(kx-\omega t\right)\right]$$

where $k(m^{-1})$ represents the wavenumber, and ω(rad/s) denotes the angular frequency. The variables $U_{1}$ and $U_{2}$, respectively, stand for the amplitudes of the fundamental wave and the second harmonic, with the following relationship established between them:

(4) $$U_{2}=\frac{\beta }{8}U_{1}^{2}k^{2}x$$

where *β*/8, *k*^2^, *x* can be uniformly regarded as describing the nonlinearity coefficient of the medium [20,27] $\beta_{0}$, facilitating subsequent DI construction.

(5) $$\beta_{0}=\frac{U_{2}}{U_{1}^{2}}$$

In actuality, the forces exerted on the surfaces of fatigue cracks are exceedingly complex, and the one-dimensional simplified model mentioned above struggles to precisely represent the nonlinear interaction process of fatigue cracks [26]. Nonetheless, the nonlinear parameters deduced from various theories can all be formulated as expressed in Equation (5). Consequently, this paper opts for the nonlinear parameter as the principal characterizing parameter for fatigue cracks and proceeds to construct the DI based on this. 

Given the presence of OSDs with varying degrees of fatigue, to facilitate the comparison of nonlinear parameters between specimens with different identifiers, the DI is further introduced as follows:

(6) $$DI=\frac{\beta_{0}\prime }{\beta_{0(o\%)}}$$

where $\beta_{0}\prime$ represents the nonlinear parameter for different OSDs under varying fatigue lives, and $\beta_{0(o\%)}\;$ denotes the nonlinear parameter for OSDs when not subjected to any load. A larger DI indicates a stronger nonlinear phenomenon of Lamb waves within the OSD. Furthermore, attempting to fit the DI and damage degree through linear or nonlinear curves is a key step in the proposed methods and is an essential part of non-destructive testing methods [8,20].

### 2.2. Experimental Specimens

**Figure 2:**
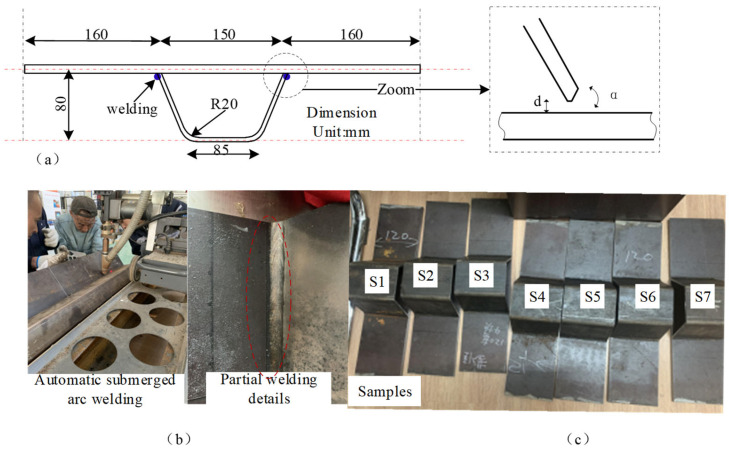
(**a**) OSD processing dimension diagram; (**b**) welding process schematic; (**c**) OSD specimen labeling diagram.

Scaled dimensions were utilized for the fabrication of the OSD. Both the top plate and U-ribs were constructed from Q345b steel, which has a density of 7850 kg/m^3^ and a Poisson’s ratio of 0.3. Material tests on the steel top plates used yielded an average elastic modulus of 218.3 GPa and an average yield strength of 400.6 MPa. The dimensions of the scaled OSD are illustrated in Figure 2a. A bevel groove weld was employed between the U-ribs and the top plate. To ensure a uniform welding quality, an automatic submerged arc welding method was used, followed by cutting. The weld penetration was no less than 75% of the plate thickness, and burn-through was not permitted. The assembly gap, b, was less than or equal to 0.5 mm, with a bevel angle of the top plate α being $50\pm 2^{0}$. After welding, a pre-fabricated crack with a depth of 1 mm and a width of less than or equal to 0.3 mm was created at the weld joint using wire cutting. The details of the top plate and U-rib welding, as well as the actual welding effect, are shown in Figure 2b. Ultimately, seven sets of specimen entities were obtained, as shown in Figure 2c, and they were respectively labeled as OSD specimens S1, S2, S3, S4, S5, S6, and S7.

### 2.3. Fatigue Loading Configuration

**Figure 3:**
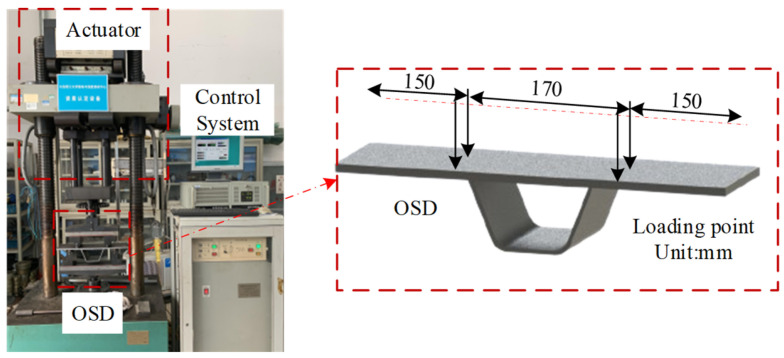
Schematic diagram of the OSD fatigue test loading process.

Fatigue loading tests were conducted at the Vibration and Strength Testing Center of Dalian University of Technology, using the PLG-200C high-frequency tension-compression fatigue testing machine, as shown in Figure 3. The tests employed a four-point bending loading method. Before setting the mean stress and stress amplitude, a simple bending normal stress test was conducted on an OSD specimen, determining the mean stress for fatigue loading to be 30 kN and the stress amplitude to be 6 kN. Under these experimental conditions, the processed and scaled OSD was subjected to fatigue loading, with the testing machine’s operating frequency being essentially stable at approximately 89.3 Hz, as depicted in Figure 3. The OSD S7 specimen was loaded until significant fracture deformation was observed, with the fatigue cycle count reaching 820,000. When noticeable deformation occurred, the testing machine’s operating frequency decreased to 88.9 Hz. Additionally, to compare the macroscopic metallographic states of the fatigue life plates, the OSD specimens S1–S6 corresponded to total cycle counts of 200,000; 300,000; 400,000; 500,000; 600,000; and 700,000, respectively. It should be noted that during the test process, all OSD specimens were paused and unloaded every 100,000 cycles for ultrasonic testing.

### 2.4. Ultrasonic Testing Configuration

**Figure 4:**
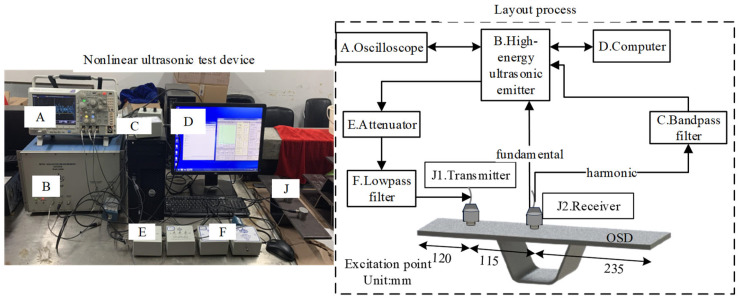
Schematic diagram of the ultrasonic testing connection for OSDs.

Figure 4 presents a schematic diagram of the ultrasonic testing process for OSDs. The excitation probe of the ultrasonic transducer is placed at the center of the plate, 120 mm from the left boundary of the top plate, while the receiving probe is positioned at the midline. Ultrasonic waves are emitted using the RITEC SNAP (model RAM-5000); then, they are passed through an attenuator and a low-pass filter to reach the transmitting probe resonating at 250 kHz. This setup is intended to better detect the second harmonic response. The receiving end employs an ultrasonic probe with a resonant frequency of 500 kHz, and the probe sends the fundamental wave signal directly back to the main system. Since the received signal’s fundamental frequency amplitude is between 10 and 100 times that of the harmonic frequency, a 500 kHz band-pass filter is set in the receiving circuit to isolate the second harmonic signal. The filters used in this study were provided by RITEC (model FDK, serial No. 9074). The low-pass filter is a 10th-order Butterworth design with a 3 dB attenuation at 250 kHz and an input impedance of 50 ohms. The band-pass filter is a 5th-order design with a 40 dB out-of-band attenuation. The excitation voltage is set at 640 V, with an attenuation of 4 dB and a gain of 40 dB. The gate amplifier is set to an output level of 32. During the nonlinear ultrasonic detection process, an oscilloscope is used to obtain the time-domain graphs of the fundamental wave signal and the second harmonic signal. The RAM-5000 SNAP system extracts the first wave signal from the time-domain graphs of both the fundamental and second harmonic signals to perform a sweep and to obtain their spectrum graphs.

**Figure 5:**
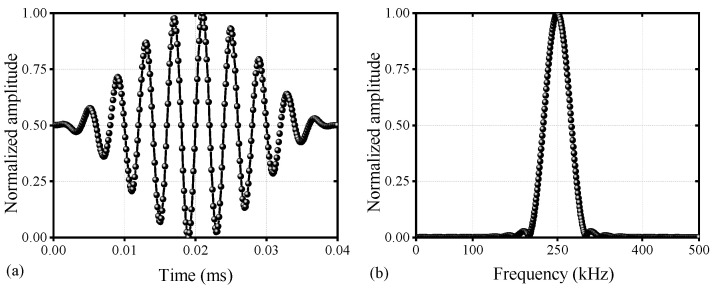
Schematic diagram of the excitation signal (**a**) time-domain and (**b**) frequency-domain signal.

As shown in Figure 1, an excitation value near 250 kHz can relatively weaken the ultrasonic frequency dispersion linearity and reduce the number of modes, matching the phase velocity with the second harmonic [28]. Using a total of 10 cycles ensures the concentration of the ultrasonic spectrum signal towards the central frequency, further weakening the dispersion effect [29]. Therefore, the excitation signal uses a ten-cycle sinusoidal modulation signal with a central frequency of 250 kHz, as shown in the following expression:

(7) $$w(t)=\left\{\begin{array}{c} 0.5\;[1-\cos(2\pi f_{c}t/n)]\sin(2\pi f_{c}t)\;\\ 0 \end{array}\begin{array}{c} t\in (0,n/f_{c})\\ otherwise \end{array}\right.$$

where w(t) is the excitation signal; $f_{c}\;(kHz)\;$ is the central frequency; and n is the number of modulation periods of the Hanning window, with its time-domain and frequency-domain expressions shown in Figure 5. The Hanning window serves to reduce the spectral leakage of the excitation signal [30,31].

**Figure 6:**
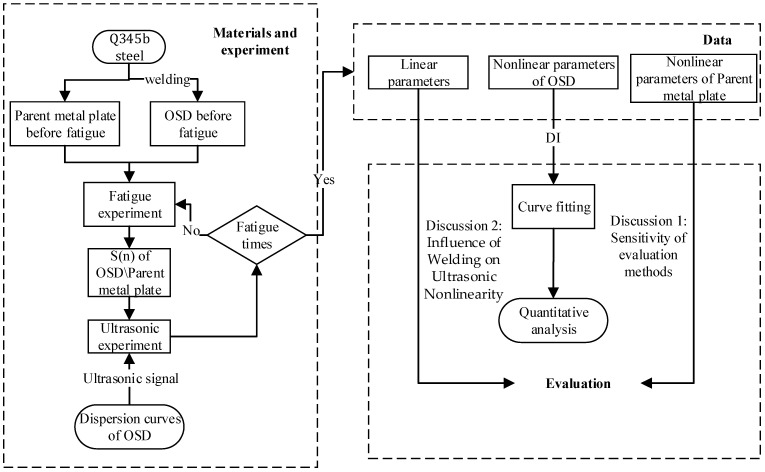
Flowchart of the evaluation method construction process for fatigue damage detection in OSDs.

To provide a more intuitive overview of the construction process of the proposed evaluation method, a flowchart is illustrated in Figure 6. This includes the materials, experimental procedures, data acquisition, quantitative analysis, and two extended discussions on the evaluation method. As shown in Figure 6, the prepared OSD specimens first undergo fatigue loading. After reaching the specified number of fatigue cycles, ultrasonic testing is performed. If the specimens do not meet the fatigue cycle requirement, fatigue testing continues after the ultrasonic test. The ultrasonic test results are presented in the form of linear and nonlinear parameters. In addition to performing nonlinear curve fitting on the seven specimens for quantitative analysis, two discussions on the evaluation method are also included. These discussions focus on the sensitivity of nonlinear ultrasonic indicators in comparison to their linear counterparts, as well as the impact of weld seams on nonlinear ultrasonic parameters in OSDs.

## 3. Results

**Figure 7:**
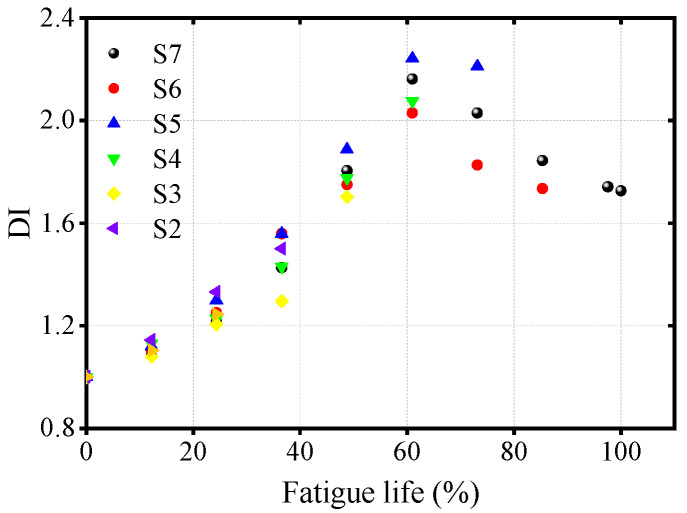
Variation of DI values for different specimens with fatigue life.

To enhance the reliability of the test data for the fundamental wave amplitude and the second harmonic amplitude, the coupling agent was reapplied for each ultrasonic monitoring session, and the test was conducted three times. The sweep amplitudes of the fundamental and second harmonics were obtained by measuring and taking the average value, which was then used to calculate the nonlinear parameter. Figure 7 summarizes the DI values for different specimens.

### 3.1. Changes in Ultrasonic Signals before and after Fatigue Loading

**Figure 8:**
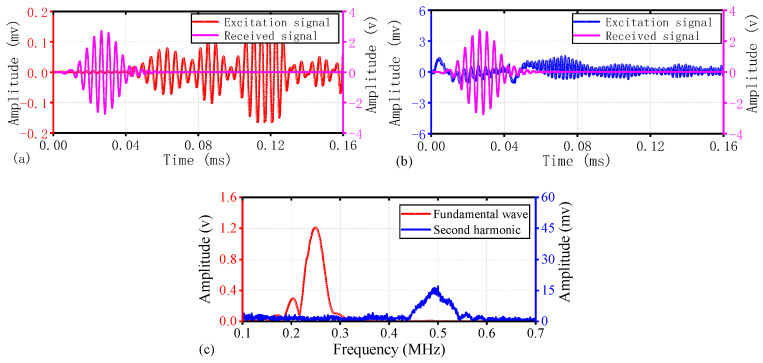
Experimental results for undamaged condition of S5: (**a**) Time-domain plot of the fundamental wave; (**b**) Time-domain plot of the harmonic wave; (**c**) Spectrogram of the fundamental and harmonic frequencies.

**Figure 9:**
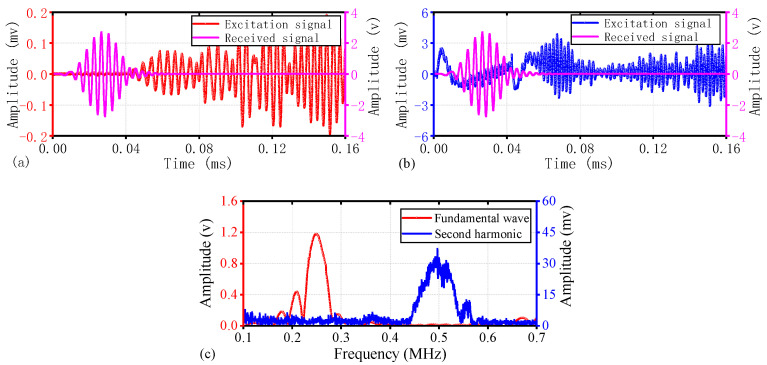
Experimental results for fatigue condition of S5: (**a**) Time-domain plot of the fundamental wave; (**b**) Time-domain plot of the harmonic wave; (**c**) Spectrogram of the fundamental and harmonic frequencies.

Due to the largest variation in the DI values of S5 (as shown in Figure 7), and since the trend of ultrasonic signal changes in other specimens is similar, this section takes S5 as an example to demonstrate the changes in the ultrasonic nonlinear effects before and after OSD fatigue loading. The fundamental wave amplitude values of S5 before and after fatigue loading are shown in Figure 8a and Figure 9a, respectively. The results indicate that the energy of the initial fundamental wave is not significantly different. The transmission times of the first wave are 0.0364 ms and 0.0403 ms, respectively, with sound speeds of 3159 m/s and 2854 m/s, respectively. The difference in sound speed is also minimal and close to the theoretical sound speed of the A0 mode in the group velocity dispersion curve shown in Figure 1 (A0: 3159 m/s), thereby confirming that the first signal to arrive is primarily in the antisymmetric mode, consistent with the excitation method of the ultrasonic signal.

Figure 8b and Figure 9b illustrate the capture of harmonic signals through a 500 kHz band-pass filter. The measurement of the harmonic wave speed is not sufficiently precise, and a rough estimate from the time-domain graph suggests a decrease in the harmonic wave speed after fatigue loading. This may be related to the apparent diffraction of the defect signal post-fatigue loading. However, the amplitude of the first wave of the second harmonic after loading was significantly increased compared to before fatigue loading, indicating that fatigue induces a strong nonlinear response in OSD. 

Figure 8c shows that before the fatigue loading of S5, the peak of the fundamental wave spectrum is around 249 kHz, while the peak of the harmonic spectrum is around 494 kHz. The harmonic frequency being a multiple of the fundamental frequency indicates that this second harmonic is a generated nonlinear signal. From the spectrum, the peak amplitude of the fundamental wave is 1.206 V, while the peak amplitude of the harmonic is 0.0154 V. The nonlinear parameter is calculated to be 0.0106; at this time, the harmonic is primarily caused by the nonlinearity of the welding structure. Post-fatigue loading, the spectrum of S5, as depicted in Figure 9c, shows the fundamental wave spectrum peak at approximately 247 kHz and the harmonic spectrum peak at around 491 kHz, with almost no change in the peak frequency values. The peak amplitude of the fundamental wave through the path of the crack is 1.171 V, while the peak amplitude of the harmonic is 0.0321 V. The nonlinear parameter is calculated to be 0.0234, indicating a significant increase due to the local nonlinearity caused by the fatigue crack. 

### 3.2. Evaluation of OSD Fatigue Life

**Figure 10:**
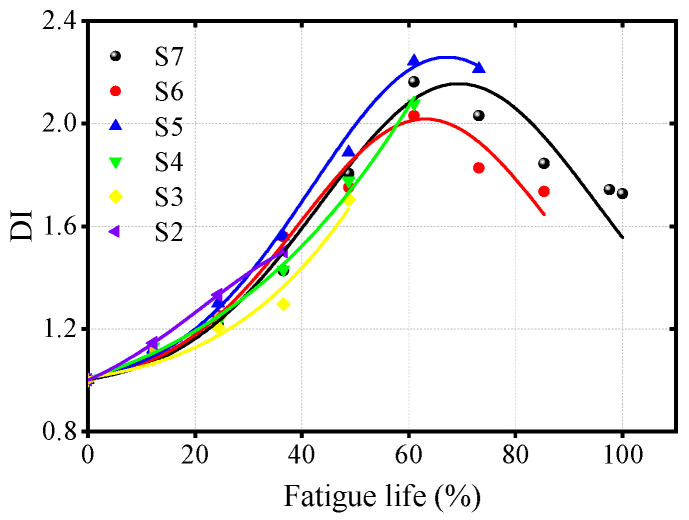
Gaussian fitting curve of OSDs’ fatigue life and DI.

Figure 10 reveals that as the fatigue cycle count increases for the seven specimens of OSDs, the DI value continuously rises, reaching a peak at approximately 61.0% of the fatigue life before subsequently decreasing. The early increase in DI may be attributed to the accumulation of dislocations at the wire-cutting sites during the initial stages of fatigue, which gradually evolve into micro-closed fatigue cracks, resulting in a ‘breathing’ phenomenon and a significant increase in nonlinear response. This process implies that the normalized nonlinear parameter can characterize the accumulation of dislocations; however, once the fatigue cracks propagate to a certain extent, the ‘breathing’ phenomenon diminishes with the widening of the crack opening, leading to a weakened nonlinear response, as indicated by a reduction in the nonlinear parameter. Additionally, it can be seen that the DI derived from nonlinear parameters does not exhibit a linear or even monotonic relationship with fatigue life. This observation is consistent with the findings of Lee et al. [20], who noted similar phenomena when analyzing fatigue cracks in steel plates using nonlinear ultrasonics. This indicates that linear or monotonic nonlinear curves are no longer applicable in this context. In contrast, a nonlinear curve based on a Gaussian model [32] can effectively represent the trend where the DI initially increases and then decreases. Therefore, to quantitatively analyze the relationship between fatigue life and DI value, a Gaussian curve is chosen for fitting, with the expression for the fitting curve as follows:

(8) $$y=y_{0}+Ae^{-\frac{{\left(x-x_{c}\right)}^{2}}{2w^{2}}}$$

where $y_{0},A,\omega ,x_{c}$ represents the shape parameters of the Gaussian function, which determine the specific form of the Gaussian curve. The numerical values of the shape parameters for the fitting curves of the specimens, other than S1, can be found in Table 1, while the correspondence between the fitting curves and the actual data is displayed in Figure 10. Due to the short fatigue loading cycle of S1, the number of data points is insufficient to satisfy the unknown values required for curve fitting, thus precluding an effective fatigue life fit for S1. However, the fitting curves for the other six groups of specimens will be validated using the data from S1.

**Figure 11:**
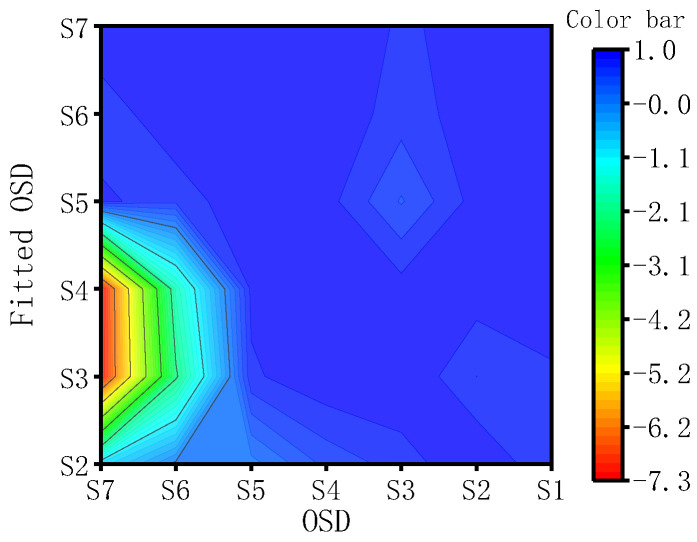
The R-squared values of the fitted curve for other OSD fittings.

Figure 11 illustrates the applicability of a single fitting curve derived from specimens S2 to S7 across all other specimens. The results indicate that for specimens experiencing fewer fatigue cycles, such as S2 to S5, the fitting curve’s applicability to other specimens is lower, with variance even reaching negative values, suggesting a suboptimal fit. In contrast, for S7, the fitting curve obtained after enduring 100% fatigue loading, when applied to other specimen groups, yielded variance values exceeding 0.81. Notably, the fitting variance for each individual specimen falls within the range of 0.97 to 1, further validating the efficacy of the Gaussian curve in predicting the relationship between fatigue life and DI in OSDs. The fitting curve extends the quantitative analysis of OSD fatigue damage assessment into the field of nonlinear ultrasonics. 

## 4. Discussion

### 4.1. Comparison with Linear Ultrasonics

**Figure 12:**
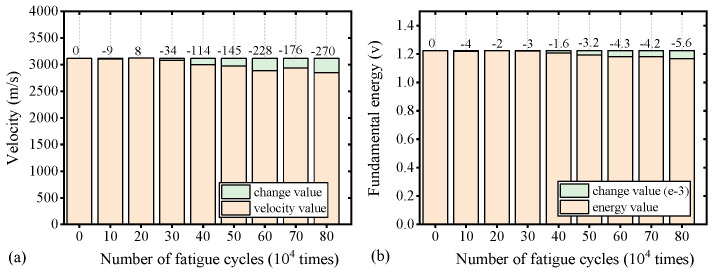
Linear ultrasonic change of S7 with different fatigue life: (**a**) velocity and (**b**) energy.

In linear ultrasonic methods, velocity and energy are commonly used metrics to assess material conditions [2,8,9]. Signal energy is calculated by squaring the amplitude of the sample signals and summing them, given that the energy density of ultrasonic waves is proportional to the square of the amplitude. This section examines changes in energy and velocity for specimen S7, as depicted in Figure 12. S7, having undergone a complete fatigue life cycle, serves as a representative data set for comparative analysis.

Figure 12 demonstrates that as fatigue cycles increase, the sound velocity and fundamental wave energy of S7 both show a slight and insignificant downward trend. The relative change rate for both parameters did not exceed 1%, suggesting a substantial potential for random error. Therefore, sound velocity and energy are not highly sensitive indicators of fatigue life and are prone to significant errors. Conversely, as shown in Figure 10, the DI based on nonlinear parameters effectively characterizes fatigue life with a broad range of variation. 

### 4.2. Influence of Welding on Ultrasonic Nonlinearity

The OSD features a U-rib structure, with the top plate being connected to the U-ribs through welding. Even advanced welding techniques cannot entirely prevent the formation of microscopic defects in the welded areas, which contribute to a nonlinear ultrasonic response. To ensure the credibility of the results, the influence of welding on ultrasonic nonlinearity must be considered. 

**Figure 13:**
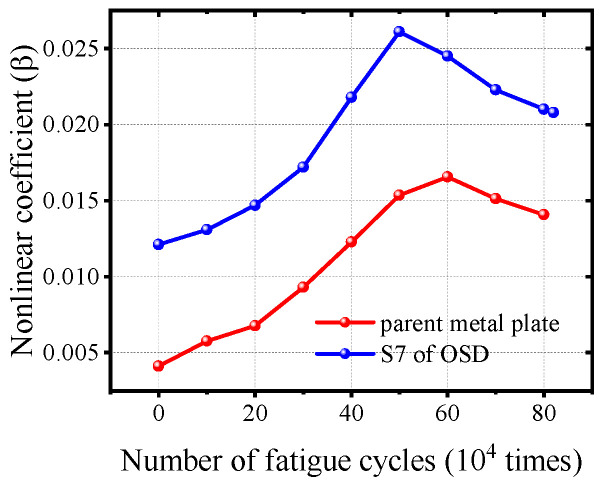
Comparison of nonlinear parameters of parent metal plate base metal and S7 before and after fatigue loading.

A parent metal plate was fabricated using 10 mm thick steel from the same batch as the OSD. Except for the welding process, the fabrication flow and ultrasonic monitoring methods were consistent with those used for the OSD preparation. Figure 13 compares the nonlinear parameters of the OSD before and after fatigue loading with those of the parent metal plate specimens. Before fatigue loading, the nonlinear parameters of the parent metal plate showed a similar trend to S7 due to fatigue damage. However, the nonlinear parameters of S7 were noticeably higher than those of the parent metal plate specimens, indicating that the welded structure within the OSD induces a significant nonlinear ultrasonic response. 

**Figure 14:**
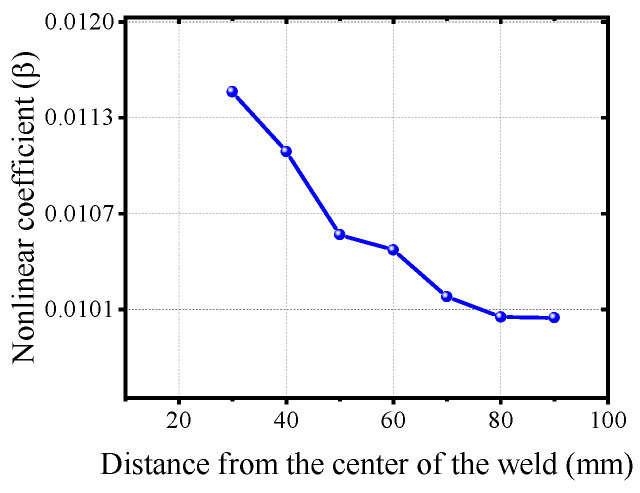
Nonlinear parameter change curve with different distance from weld.

Furthermore, prior to fatigue loading, the excitation probe was placed at varying distances from the left end of the weld seam on seven sets of OSD specimens. The receiving probe was correspondingly moved to maintain a total transmission distance of 115 mm for testing to investigate the weld seam’s impact on the nonlinear parameters. Figure 14 illustrates the average nonlinear parameters at different distances from the weld seam. It shows that the closer the excitation probe is to the center of the weld seam, the greater the nonlinear parameters and the more pronounced the nonlinear response is. This suggests that the welded structure produces a certain degree of nonlinear response.

As the excitation probe moves further from the center of the weld seam, the nonlinear parameters gradually decrease. However, when the distance becomes sufficiently large, the change in nonlinear parameters is minimal. This indicates that part of the nonlinear response in the OSD fatigue damage test is due to the weld seam. Therefore, ultrasonic detection results should not be attributed solely to fatigue loading, as this may introduce a degree of error. This influence is also why different Gaussian curve shape parameters were observed among the seven sets of OSD specimens. Unfortunately, due to the presence of weld seams, the proposed method cannot achieve a baseline-free characteristic, which poses challenges for assessing existing bridges. Nonetheless, it remains suitable for evaluating newly constructed bridges. 

## 5. Conclusions

This study proposes a method for detecting the fatigue life of OSDs based on nonlinear ultrasonic parameters. By employing a nonlinear ultrasonic system, tests were conducted on seven OSD specimens and a parent plate. It was observed that in the early stages of fatigue, the nonlinear effects in the OSDs significantly increased, peaking at 61% of the fatigue life, after which they began to decline. Concurrently, the study compared the performance of linear ultrasonic indicators in assessing OSDs’ fatigue life and found no significant changes under fatigue loading. In contrast, the DI based on nonlinear parameters could be well-fitted to the remaining fatigue life of OSDs using a Gaussian curve, enabling the quantitative analysis of OSDs’ fatigue life. Additionally, the change trend of the nonlinear parameters of OSDs was consistent with the increase in fatigue life when compared to steel plates of the same thickness, confirming that weld seams do not affect the effectiveness of the nonlinear ultrasonic method in evaluating fatigue life. This research lays the groundwork for the practical application of nonlinear ultrasonic parameters in predicting the fatigue life of OSDs.

However, the nonlinear response induced by the welded structures within the OSDs leads to variations in the fitting curve parameters among different specimens. This nonlinear response from the weld seams must be considered in ultrasonic testing to avoid misinterpreting the results as solely being caused by fatigue loading. This limitation prevents the evaluation method from being baseline-free, thereby constraining the assessment of existing bridges. Addressing this issue remains a focal point for future research.

## Figures and Tables

**Table 1:** Parameter values of different OSD fitting curves.

Specimens	$y_{0}$	A	ω	$x_{c}$
S2	0.97042	0.69347	0.25813	1.18431
S3	0.97996	0.63097	0.23621	1.03675
S4	0.97254	0.67169	0.25296	1.28564
S5	0.83359	2.12618	0.74694	9.94756
S6	0.96443	1.24339	0.42064	3.52888
S7	0.86297	0.48607	0.26865	0.70411

## Data Availability

The data presented in this study are available on request from the corresponding author.

## References

[1] Kozy B.M., Connor R.J., Paterson D., Mertz D.R. (2011). Proposed Revisions to Aashto-Lrfd Bridge Design Specifications for Orthotropic Steel Deck Bridges. J. Bridge Eng..

[2] Shi L., Cheng B., Xiang S., Li D., Liu T. (2023). Monitoring for Fatigue Crack Geometry in Orthotropic Steel Bridge Decks by Application of Reflected Lamb Waves. Thin-Walled Struct..

[3] Zeng Y., He H., Qu Y., Sun X., Tan H., Zhou J. (2023). Numerical Simulation of Fatigue Cracking of Diaphragm Notch in Orthotropic Steel Deck Model. Materials.

[4] Liu Y., Huang W., Yu B., Chen Z., Wang P. (2023). Fatigue Performance Analysis of Welded T-Joints in Orthotropic Steel Bridge Decks with Ultrasonic Impact Treatment. Materials.

[5] Abdelbaset H., Zhu Z. (2024). Behavior and Fatigue Life Assessment of Orthotropic Steel Decks: A State-of-the-Art-Review. Structures.

[6] Fisher J.W., Barsom J.M. (2016). Evaluation of Cracking in the Rib-to-Deck Welds of the Bronx–Whitestone Bridge. J. Bridge Eng..

[7] Tecchio G., Lorenzoni F., Caldon M., Donà M., da Porto F., Modena C. (2017). Monitoring of Orthotropic Steel Decks for Experimental Evaluation of Residual Fatigue Life. J. Civ. Struct. Health Monit..

[8] Gao S., Zhang R., Fan Z., Li N., Yue Y., Xie L. (2024). Online Monitoring of Fatigue Damage in Welded Joints Using Diffuse Ultrasound. Ultrasonics.

[9] Pahlavan L., Blacquière G. (2016). Fatigue Crack Sizing in Steel Bridge Decks Using Ultrasonic Guided Waves. NDT E Int..

[10] Shi L., Cheng B., Li D., Xiang S., Liu T., Zhao Q. (2023). A Cnn-Based Lamb Wave Processing Model for Field Monitoring of Fatigue Cracks in Orthotropic Steel Bridge Decks. Structures.

[11] Deng Y., Li A., Feng D. (2018). Fatigue Reliability Assessment for Orthotropic Steel Decks Based on Long-Term Strain Monitoring. Sensors.

[12] Di J., Ruan X., Zhou X., Wang J., Peng X. (2021). Fatigue Assessment of Orthotropic Steel Bridge Decks Based on Strain Monitoring Data. Eng. Struct..

[13] Wei S., Zhang Z., Li S., Li H. (2017). Strain Features and Condition Assessment of Orthotropic Steel Deck Cable-Supported Bridges Subjected to Vehicle Loads by Using Dense Fbg Strain Sensors. Smart Mater. Struct..

[14] Li D., Nie J.-H., Ren W.-X., Ng W.-H., Wang G.-H., Wang Y. (2022). A Novel Acoustic Emission Source Location Method for Crack Monitoring of Orthotropic Steel Plates. Eng. Struct..

[15] Sakagami T. (2015). Remote Nondestructive Evaluation Technique Using Infrared Thermography for Fatigue Cracks in Steel Bridges. Fatigue Fract. Eng. Mater. Struct..

[16] Yamada T., Shiraishi A., Okuno M., Sugiyama H., Kanjo N., Tsukamoto S., Yamagami T. Application of Electromagnetic Testing to Orthotropic Steel Deck. Proceedings of the 6th International Conference on Bridge Maintenance, Safety and Management.

[17] Farreras-Alcover I., Chryssanthopoulos M.K., Andersen J.E. (2017). Data-Based Models for Fatigue Reliability of Orthotropic Steel Bridge Decks Based on Temperature, Traffic and Strain Monitoring. Int. J. Fatigue.

[18] Liu J., Li T., Li D., Shen W. (2024). Experimental and Numerical Validation of Guided Wave Based on Time-Reversal for Evaluating Grouting Defects of Multi-Interface Sleeve. Smart Struct. Syst..

[19] Bjørheim F., Siriwardane S.C., Pavlou D. (2022). A Review of Fatigue Damage Detection and Measurement Techniques. Int. J. Fatigue.

[20] Lee Y.F., Lu Y. (2022). Advanced Numerical Simulations Considering Crack Orientation for Fatigue Damage Quantification Using Nonlinear Guided Waves. Ultrasonics.

[21] Hu B., Amjad U., Kundu T. (2024). Monitoring Fatigue Cracks in Riveted Plates Using a Sideband Intensity Based Nonlinear Ultrasonic Technique. Ultrasonics.

[22] Wang X., Wang X., Niu X.-G., Xiao D.-M., Hu X.-L. (2018). Application of Nonlinear Ultrasonic Technique to Characterize the Creep Damage in Asme T92 Steel Welded Joints. NDT E Int..

[23] Worlton D.C. (1961). Experimental Confirmation of Lamb Waves at Megacycle Frequencies. J. Appl. Phys..

[24] Zhang H., Wang F., Lin J., Hua J. (2024). Lamb Wave-Based Damage Assessment for Composite Laminates Using a Deep Learning Approach. Ultrasonics.

[25] Chen H., Zeng J., Wang J., Xu B., Mo Y. (2023). Multiscale Homogenization Numerical Study on the Mechanism of Interface Debonding Detection for Steel–Concrete Composite Structures with Multichannel Surface Wave Measurements. Constr. Build. Mater..

[26] Wang K., Liu M., Su Z., Yuan S., Fan Z. (2018). Analytical Insight into “Breathing” Crack-Induced Acoustic Nonlinearity with an Application to Quantitative Evaluation of Contact Cracks. Ultrasonics.

[27] Nilsson M., Huttunen-Saarivirta E., Bohner E., Ferreira M. (2023). Non-Destructive Evaluation of Corrosion in Steel Liner Plates Embedded in Concrete Using Nonlinear Ultrasonics. Constr. Build. Mater..

[28] Kanakambaran K.V., Balasubramaniam K. (2024). Frequency Sweep Study on the Generation of Dual-Mode Second Harmonics (Dmsh) on an Isotropic Nonlinear Elastic Cylindrical Rod by T(0,1) Mode. J. Sound Vib..

[29] Wang K., Liu M., Su Z., Guo S., Cui F. (2021). Mode-Mismatching Enhanced Disbond Detection Using Material Nonlinearity in Guided Waves at Low Frequency. J. Sound Vib..

[30] Asokkumar A., Jasiūnienė E., Raišutis R., Kažys R.J. (2021). Comparison of Ultrasonic Non-Contact Air-Coupled Techniques for Characterization of Impact-Type Defects in Pultruded Gfrp Composites. Materials.

[31] Liu J., Jun Y., Li D., Cui X., Zhou J. (2023). Combined Two-Level Guided Wave Structural Health Monitoring Strategy Using Multifeature Integration and Machine Learning: Application to Early-Age Grouted Sleeves. Smart Mater. Struct..

[32] Motulsky H.J., Lennart A.R. (1987). Fitting Curves to Data Using Nonlinear Regression: A Practical and Nonmathematical Review. FASEB J..

